# Country-specific regulation and international standardization of cell-based therapeutic products derived from pluripotent stem cells

**DOI:** 10.1016/j.stemcr.2023.05.003

**Published:** 2023-08-08

**Authors:** Takamasa Hirai, Satoshi Yasuda, Akihiro Umezawa, Yoji Sato

**Affiliations:** 1 Division of Cell-Based Therapeutic Products, National Institute of Health Sciences, Kanagawa, Japan; 2 Center for Regenerative Medicine, National Center for Child Health and Development, Tokyo, Japan

## Abstract

Currently, many types of cell-based therapeutic products (CTPs) derived from pluripotent stem cells (PSCs) are being developed in a lot of countries, some of which are in clinical trial stages. CTPs are classified differently in different countries and regions. The evaluation of their efficacy, safety, and quality also differs from that for conventional small-molecule drugs and biopharmaceuticals, which reflects the complex properties of living cells and unmet medical needs. Since there are no international guidelines to evaluate CTPs, including PSC-derived products, it is necessary to be aware of differences in relevant laws and regulations in different countries and regions. International consortia are organized and actively working to standardize/harmonize the evaluation methods and regulations to facilitate the development and global distribution of PSC-derived CTPs. In this paper, we outline the regulations related to PSC-derived CTPs in the International Council for Harmonization of Technical Requirements for Pharmaceuticals for Human Use founding regions (US, EU/UK, Japan) and introduce representative consortia working on their standardization.

## Teaser

This review outlines the current regulations related to pluripotent stem cell-derived therapeutic products in the United States, the European Union (and the United Kingdom), and Japan and introduces representative consortia working on their standardization.

## Introduction

Embryonic stem cells (ESCs) and induced pluripotent stem cells (iPSCs) are types of pluripotent stem cells (PSCs) capable of infinite self-renewal and differentiation into different cell types. By exploiting the characteristics of PSCs, target cells can be differentiated from human ESCs/iPSCs and used as therapeutic products for intractable diseases in regenerative medicine and cell therapy. Cell-based therapeutic products (CTPs), including PSC-derived products, are different from small-molecule and antibody drugs and are also commonly regarded as innovative pharmaceuticals. As CTPs are composed of living cells and vary widely in raw materials, manufacturing processes, final product form, and disease treatment, a case-by-case approach should be applied to each product to ensure its quality and safety. Therefore, conventional test methods and good practices applied to pharmaceuticals are often not applicable to CTPs. Furthermore, there is no international consensus on the evaluation and good practices related to the efficacy, safety, and quality of CTPs, and no internationally available regulatory guidelines have been issued.

In the United States (US), the European Union (EU) (and the United Kingdom [UK]), and Japan, the three founding members of the International Council for Harmonization of Technical Requirements for Pharmaceuticals for Human Use (ICH), regulations are established according to the product categories defined in each country and region. CTPs are usually classified according to whether they contain manipulated/processed cells or whether they are intended for homologous use. In the US, therapeutic products containing cells with more-than-minimal manipulation, including PSC-derived products, are deemed human cells, tissues, and cellular and tissue-based products under Section 351 of the Public Health Service Act (351 HCT/Ps) and classified as biological products or medical devices, whereas CTPs containing substantially manipulated/processed cells are classified as advanced therapy medicinal products (ATMPs) and regenerative medical products in the EU (and the UK) and Japan, respectively. CTPs are approved for marketing after undergoing a review process that is unique to each country or region. Also, clinical trials using CTP involving transplantation or surgical procedures must comply with national regulations regarding ethics. In addition, the rare and serious disease areas that are often the targets of CTPs often do not have sufficient patient populations for their clinical development. For these reasons, time is required to collect and evaluate data for efficacy validation, and thus, each country or region has its own system for the early approval of CTPs (Sato et al., 2019; Yoneda et al., 2021). In this current situation, standardization of manufacturing and evaluation techniques and harmonization/convergence of regulations are effective approaches to accelerate the product development and expedite approval. Discussions are currently being held on a wide variety of topics related to regenerative medicine, including definitions of terms, cell sampling and storage methods, manufacturing processes, and evaluation tests by various international consortia that collaborate with each other.

This paper describes the following: (1) the review systems, regulations, and guidelines related to the approval process for CTPs, including PSC-derived products, in the US, EU, and Japan; (2) expedited approval systems in each country and region that were developed in consideration of the characteristics of CTPs and unmet medical needs; and (3) representative international platforms working on the technical standardization or regulatory harmonization/convergence of CTPs.

### The principle of regulation: A risk-based approach

The risk-based approach is a general principle for the regulation of pharmaceutical products in the three ICH founding states and has also been adopted in the ICH Guidance on Quality Risk Management (Q9). The risk-based approach establishes regulatory policies and content by scientifically evaluating the degree of impact of a product, based on the identification of risk factors inherent to the property of each product with respect to efficacy, safety, and quality (Table 1, #1, #13, #17–19) (FDA/CBER, 1997; EMA/CAT/CPWP, 2013; MHLW/PFSB, 2012a, 2012b, 2012c; Hayakawa et al., 2015a, 2015b, 2015c). It is difficult to achieve a uniform quality of CTPs, including PSC-derived products, owing to various factors such as differences in cell donors, as well as complexity and activity of cells as living organisms. In addition, raw materials, manufacturing processes, final product forms, and clinical use vary widely from product to product. Therefore, the quality profiles and uses of final products are highly diverse, and the associated risk factors and risk severity vary among the products. To obtain a risk profile, it is necessary to scientifically evaluate the risk factors for each product from an early stage of development.

**Table 1:** Regulatory guidelines related to cell-based therapeutic products

Agency	Guidelines	Relevant laws and regulations
FDA	1. Proposed approach to regulation of cellular and tissue-based products (docket number 97N-0068) 2. Eligibility determination for donors of human cells, tissues, and cellular and tissue-based products (docket number 2004D-0193) 3. Potency tests for cellular and gene therapy products (docket number FDA-2008-D-0520) 4. **CTGTAC. Cellular therapies derived from human embryonic stem cells: considerations for preclinical safety testing and patient monitoring. Meeting # 45 (briefing document, not guidance for industry)** 5. Preclinical assessment of investigational cellular and gene therapy products (docket number FDA-2012-D-1038) 6. Considerations for the design of early-phase clinical trials of cellular and gene therapy products (docket Number FDA-2013-D-0576) 7. Regulatory considerations for human cells, tissues, and cellular and tissue-based products: minimal manipulation and homologous use (docket number FDA-2017-D-6146) 8. Regulation of human cells, tissues, and cellular and tissue-based products (HCT/Ps) – small entity compliance guide (docket number: FDA-2022-D-0563) 9. Voluntary consensus standards recognition program for regenerative medicine therapies (docket number: FDA-2022-D-0745)	a. Federal Food, Drug, and Cosmetic Act b. Public Health Service Act c. 21^st^ Century Cures Act (Public Law 114–255) d. Code of Federal Regulations Title 21 (21CFR) Part 1271
EMA	10. Guideline on human cell-based medicinal products (EMEA/CHMP/410869/2006) 11. Guideline on safety and efficacy follow-up and risk management of advanced therapy medicinal products (EMEA/149995/2008) 12. **Reflection paper on stem cell-based medicinal products (EMA/CAT/571134/2009)** 13. Guideline on the risk-based approach according to annex I, part IV of Directive 2001/83/EC applied to advanced therapy medicinal products (EMA/CAT/CPWP/686637/2011) 14. Reflection paper on a proposal to enhance early dialogue to facilitate accelerated assessment of priority medicines (PRIME) Draft (EMA/CHMP/57760/2015) 15. Guidelines on Good Manufacturing Practice specific to Advanced Therapy Medicinal Products [C(2017) 7694 final] 16. Guideline on Good Clinical Practice specific to Advanced Therapy Medicinal Products [C(2019) 7140 final]	d. REGULATION (EC) No 1394/2007 on Advanced Therapy Medicinal Products e. Directive 2009/120/EC
MHLW	17. **Guidelines on ensuring the quality and safety of pharmaceuticals and medical devices derived from the processing of autologous human iPS(-like) cells (Notifications No. 0907-4, PFSB) (2012)** 18. **Guidelines on ensuring the quality and safety of pharmaceuticals and medical devices derived from the processing of allogeneic human iPS(-like) cells (Notifications No. 0907-5, PFSB) (2012)** 19. **Guidelines on ensuring the quality and safety of pharmaceuticals and medical devices derived from the processing of allogeneic human ES cells (Notifications No. 0907-6, PFSB) (2012)** 20. **Evaluation guidelines for autologous iPS cell-derived retinal pigment epithelial cells (Notification No. 0529-1, Attachment 1, PFSB/ELD/OMDE) (2013)** 21. **Evaluation guidelines for allogeneic iPS cell-derived retinal pigment epithelial cells (Notification No. 0912-2, Attachment 1, PFSB/ELD/OMDE) (2014)** 22. **Technical guidance for quality, non-clinical and clinical studies of regenerative medicine products (human cell-processed products) (PSEHB/MDED Administrative Notice No. 0614043) (2016)** 23. **Evaluation guidelines for articular cartilage regeneration using allogeneic iPS (like) cell-processed products (Notification No. 0630-1, Attachment 2, PSEHB/MDED) (2016)** 24. **Points to consider regarding tests to detect undifferentiated pluripotent stem cells/transformed cells, tumorigenicity tests, and genomic stability evaluation for human cell-based therapeutic products (Notification No. 0627-1, PSEHB/MDED) (2019)** 25. **Evaluation guidelines for the treatment of (traumatic) subacute spinal cord injury using human (allogeneic) iPS (like) cell-processed products (Notification No. 0226-1, PSEHB/MDED) (2021)** 26. **Points for certified special committees for regenerative medicine to consider when evaluating tumorigenicity assessment in provision plans of regenerative medicine using human pluripotent stem cells (Notifications No. 0309-1, HPB/RDD) (2021)** 27. **Evaluation guidelines for the treatment of ischemia cardiomyopathy using human (allogeneic) iPS cell-derived cardiomyocyte cell-sheet (Notification No. 0331-15, PSEHB/MDED) (2023)**	f. Pharmaceuticals and Medical Devices Act g. Act on the Safety of Regenerative Medicine

Bold: document with specific descriptions about pluripotent stem cell-derived products.

### Regional regulations of PSC-derived CTPs

#### The United States

##### Product classification in the US

Cells, tissues, and cell/tissue processed products are called 361 HCT/Ps if they meet all the requirements presented in Section 361 of the of the Public Health Service Act, such as that the cells are not subjected to more than minimal manipulation and that the product is intended for homologous use only. When none of these are the case, the products are referred to as 351 HCT/Ps. Minimal manipulation means processing that does not alter the relevant biological characteristics of the cells or tissues. PSC-derived CTPs are therefore considered to be 351 HCT/Ps as their manufacturing process is not regarded to involve “minimal manipulation” but “more than minimal manipulation” (Table 1, #1 and #7) (FDA/CBER, 1997; FDA/CBER/CDRH/OCP, 2020; Federal Food, Drug, and Cosmetic Act, 1938). Based on the primary mode of action principle, 351 HCT/Ps are classified as either biologics (pharmacological, immunological, and/or metabolic effects) or medical devices (structural and/or physical effects). In the case of CTPs, which are difficult to classify as biologics or medical devices, the Food and Drug Administration’s (FDA’s) Office of Combination Products (OCP) determines the classification.

##### Clinical trials

Any clinical trials for 351 HCT/Ps must be conducted in compliance with the ICH Good Clinical Practice (GCP) guidelines. In addition, following the applications, biological product: Investigational New Drug (IND) and medical device: Investigational Device Exemption from the FDA, approval is needed to conduct clinical trials for 351 HCT/Ps. For the practical application of 351 HCT/Ps, developers are entitled to receive support, for example, through a program that allows them to consult the FDA.

##### Expedited marketing authorization system in the US

It is necessary to apply for a Biologics License Application or a Premarket Approval after clinical trials to obtain marketing authorization for HCT/Ps. The FDA has established the Office of Cell, Tissue and Gene Therapy (OCTGC) of the Center for Biologics Evaluation and Research (CBER) as the point of contact for reviewing 351 HCTP/s, regardless of whether they are a biologic or a medical device.

The 21^st^ Century Cures Act was established in 2016 with the primary objective of addressing unmet medical needs and incorporating the patient’s perspective into the regulation of drugs and medical devices, building 21st century medical care, promoting rational clinical trials, supporting continued innovation by government agencies, and reforming the regulatory process. The content related to regenerative medicine is described in Section 3033–3036 of the Act. As described in Section 3033 of the “Accelerated Approval for Regenerative Medicine Advanced Therapies,” the Regenerative Medicine Advanced Therapies (RMAT) designation, which enables accelerated approval of HCT/Ps, including PSC-derived products, was stipulated. The requirements for RMAT are as follows: (1) the product is intended to be a regenerative therapy; (2) the product is intended to treat, correct, repair, or cure a serious or life-threatening disease or condition; or (3) preliminary clinical results indicate that the product has the potential to address an unmet medical need for such diseases or conditions. Priority review or accelerated approval may be applied if the RMAT designation is granted after submitting an IND application. Priority review is a program that reduces the review period from the usual 10 months to approximately 6 months (Food and Drug Administration Modernization Act of 1997, 1997). In addition, accelerated approval is a program under which marketing authorization of a drug may be granted for a serious or fatal disease if a well-controlled clinical trial demonstrates efficacy for a surrogate endpoint or for an endpoint other than survival or irreversible conditions (Code of Federal Regulations Title 21, 21 CFR). Products designated as RMAT are eligible for consultations regarding the surrogate/intermediate endpoint for Accelerated Approval. Further clinical trials are required to confirm the efficacy of the product. Approval for the product is revoked if the FDA determines that the efficacy of the product is inadequate, based on the results of post-marketing surveillance reports. In contrast, if the FDA determines that the product is effective, the obligation for post-marketing surveillance ends.

HCT/Ps classified as medical devices are eligible for humanitarian device exemption (HDE) under the humanitarian use device (HUD) designation program. An HUD is defined as a medical device that is beneficial to the patient in the treatment or diagnosis of a disease or condition that affects or develops in 4,000 or fewer people per year in the US, for which no other effective devices are available. An HUD is designated by the Office of Orphan Product Development of the FDA. Once an HDE is granted to an HUD-designated medical device, efficacy data may be substituted to explain its probable benefit during the regulatory review process. Therefore, it is not necessary to submit the results of clinical studies that scientifically demonstrate the efficacy of the device. However, it should be noted that medical devices approved under an HDE must be approved by the Institutional Review Board prior to use at medical institutions (Code of Federal Regulations Title 21).

##### Guidelines for evaluating efficacy, safety, and quality

In the US, although no specific guidance for PSC-derived CTPs with clinical application has been issued by regulatory bodies, several guidelines for HCT/Ps are available. ESCs and iPSCs can be the raw materials for allogeneic PSC-derived CTPs. As virus clearance during the manufacturing process of CTPs is impossible, the eligibility of the donor is very important particularly in the case of allogeneic products. In the “Eligibility Determination for Donors of Human Cells, Tissues, and Cellular and Tissue-Based Products (HCT/Ps)” (FDA/CBER, 2007) (Table 1, #2), information on donor eligibility testing and communicable disease agents are described in 21 CFR Part 1271.3(r) (Code of Federal Regulations Title 21). As with general pharmaceuticals, the efficacy, safety, and quality of CTPs is verified during preclinical studies; however, the intrinsic material composition and putative MOAs differ from those of small-molecule drugs, biopharmaceuticals, and medical devices. Therefore, the traditional approach used in preclinical toxicity testing is often inappropriate for evaluating the safety of CTPs. With CTPs, it is necessary to consider the impacts of biologically active substances secreted from cells and contamination with tumorigenic cells, including transformed cells and undifferentiated cells. In addition, if there is a possibility that non-cellular components and/or impurities originating during the manufacturing process are present in CTPs, their toxicity should be evaluated. The Preclinical Assessment of Investigational Cellular and Gene Therapy Products is issued by the CBER and OCTGT, and it provides information about the design of preclinical studies for HCT/Ps (e.g., animal species selection, proof-of-concept studies, and toxicology studies) (Table 1, #5) (FDA/CBER, 2013). The evaluation of CTPs is influenced by product origin, such as donor and tissue sources, level of manipulation, stage of differentiation at the time of administration, cellular heterogeneity, and batch variability. In addition, the characteristics of CTPs, such as engraftment at the site of administration or migration to other sites, can be affected by the surrounding microenvironment because they have unique complexities because of the dynamic nature of living cells. This also affects teratoma/tumor formation due to the residual undifferentiated PSCs and a range of different transformed cell types arising as a result of genetic variations, which is a concern for PSC-derived products. Therefore, the quality and efficacy can affect the fate of the product after administration, including its distribution, differentiation, integration, and tumorigenicity, and should be verified in preclinical studies. “The Considerations for the Design of Early-Phase Clinical Trials of Cellular and Gene Therapy Products” (FDA/CBER, 2015) (Table 1, #6) states that an early-phase clinical trial design should include dosimetry, a feasibility assessment, and an activity assessment. The objectives of early-phase trials are to assess the risks of the trial and protect the subjects. In particular, the priority of first-in-human trials is to evaluate safety, which includes an assessment of the nature and frequency of potential adverse effects and an estimation of their relationship to the dosage (Code of Federal Regulations Title 21). Potency assays are required for biological products, and CGT applications are also described in the document “Potency Tests for Cellular and Gene Therapy Products” (FDA/CBER, 2011) (Table 1, #3). However, the manufacturing process of HCT/Ps has a significant impact on efficacy, safety, and quality. The manufacture of 351 HCT/Ps as biologics and medical devices requires compliance with current Good Manufacturing Practice (cGMP) and the current quality system regulation (QSR), respectively, as well as the current Good Tissue Practice (cGTP) (Code of Federal Regulations Title 21). These documents provide good practices for standard operating procedures, process validation, and instrument calibration/validation to maintain a sterile manufacturing environment. A unique aspect of the cGTP is that it is based on preventing the spread of infectious diseases, as the products are human cells and tissues. To help better understand the relevant guidelines, a Q&A style guidance document has been published (Table 1, #8) (FDA/CBER, 2022a).

##### Recommendations to the FDA by the Cellular, Tissue, and Gene Therapies Advisory Committee

The Cellular, Tissue, and Gene Therapies Advisory Committee (CTGTAC) is an advisory committee of the FDA, and its expert members review and evaluate data on the efficacy, safety, and appropriate use of human cells, tissues, and other materials for regenerative medicine. The results of their discussions are then used as recommendations for the FDA. Cellular therapies derived from human ESCs were discussed at the CTGTAC Meeting #45 in 2008, and they then mainly summarized the study designs that avoid adverse events that are mainly attributable to the teratoma-forming potential of ESCs (Table 1, #4) (FDA Cellular, Tissue, and Gene Therapies Advisory Committee, 2008). To manufacture human ESC-derived CTPs, it is important to minimize the number of undifferentiated human ESCs. Tests used to detect unacceptable amounts of undifferentiated human ESCs and other cellular impurities are required to evaluate the quality of human ESC-derived CTPs. In addition, the selection of animal species and models, cell dose, and site of administration (niche) should be considered during preclinical studies to assess the potential for tumorigenesis and inappropriate differentiation at nontarget sites. These factors significantly affect the efficacy and safety of the product, as they are involved in cell engraftment, differentiation, and migration. These requirements also apply to clinical studies. Clinical trials using human ESC-derived CTPs should be conducted in patients with the target disease. In clinical trials, non-invasive techniques, such as imaging and blood markers should be used to detect potential adverse events, including the formation of teratomas and other tumors. However, key parameters for clinical trials must be carefully chosen because early signs of adverse events may be undetectable. Appropriate study designs should be established based on scientific evidence.

Although the opinion of the CTGTAC with respect to human ESC-derived CTPs could be applicable to iPSC-derived CTPs, as far as we know, no CTGTAC meeting discussions have focused on iPSC-derived CTPs. Different from ESCs, iPSCs can be generated by the reprogramming of somatic cells and clinically used for autologous transplantation. Additional discussions about the clinical application of iPSC-derived products are expected.

##### Standardization of relevant technologies in the US

Section 3036 of the 21^st^ Century Cures Act “Standards for Regenerative Medicine and Regenerative Advanced Therapies” states that efforts shall be made to formulate the definition of terminology and standards to support the development, evaluation, and review of regenerative medicine therapies/advanced regenerative therapies, including product manufacturing processes and controls. This formulation is based on regulations made through public processes with the National Institute of Standards and Technology (NIST). The Standards Coordinating Body (SCB) is a new consortium established under the leadership of the Science and Technology Committee for the Alliance for Regenerative Medicine (ARM). The SCB, in partnership with the NIST and FDA, contributes to international standardization activities in cooperation with industry associations. For example, the members of SCB, as well as groups such as FDA and standards committee members from various countries, join in the International Organization for Standardization (ISO) Technical Committee (TC) 276, which is discussed below.

The policy states that the preferential usage of standards, which are internationally harmonized and consistent with US law, increases the availability of reviews and leads to a shortened time-to-market period. For the purpose of facilitating the development and assessment of regenerative medicine therapy products, the FDA’s CBER has recently issued a draft guidance document on the use of voluntary consensus standards for regenerative medicine therapies (Table 1, #9) (FDA/CBER, 2022b). This guideline describes the requirements that need to be met in the process of developing voluntary consensus standards, with respect to how standards are recognized and how they will be reviewed for recognition.

#### The European Union

##### Product classification

In the EU, CTPs, including PSC-derived products, are handled as ATMPs, which require regulatory approval (The European Parliament and the Council of the European Union, 2007). ATMPs are human medicines based on genes, tissues, or cells that offer groundbreaking new opportunities for the treatment of diseases and injuries. ATMPs can be classified into three main types: gene therapy medicines, somatic-cell therapy medicines, and tissue-engineered medicines. The classification of somatic-cell therapy medicines and tissue-engineered medicines is based on the principle of the primary mode of action. Namely, the expected functions of somatic-cell therapy medicines and tissue-engineered medicines are “pharmacological, immunological, or metabolic” and “structural or physical,” respectively (Table 1, #12) (European Medicines Agency (a); EMA/CAT, 2011). In scientific recommendations of EMA, retinal pigment epithelial cells derived from iPSCs were classified as tissue-engineered products (European Medicines Agency, 2014). For the handling of ATMPs in the UK, which has left the EU, please refer to the guidance that is described by Cell and Gene Therapy Catapult (Cell and Gene Therapy Catapult, 2021).

##### Clinical trials in the EU

The Clinical Trials Regulation (The European Parliament and the Council of the European Union, 2014) was established in place of the Clinical Trials Directive (The European Parliament and the Council of the European Union, 2001a) to protect the right, safety, dignity, and well-being of subjects in clinical trials, and it applies to all clinical trials that involve the use of medicinal products for humans in the EU. The aim of the Clinical Trials Regulation is to harmonize the processes for assessing and supervising clinical trials. To date, the evaluation, authorization, and supervision of clinical trials are the responsibilities of EU Member States and European Economic Area countries (The European Parliament and the Council of the European Union, 2001a). Manufacturers of medicinal products for human use had to submit clinical trial applications separately to the national competent authorities and ethics committees in each country to run a clinical trial. Therefore, even if the same clinical trial applications are submitted to different member countries, the conclusions may differ from country to country. To obtain approval to conduct a clinical trial in up to 30 EU/EEA countries, the Clinical Trials Regulation, which entered into application on 31 January 2022, allows manufacturers to simultaneously submit online applications using the same documentation via the Clinical Trials Information System (CTIS) (European Medicines Agency (b)). When applying for multinational trials, all concerned Member States of the EU share any considerations and coordinately review the application of Part I, while each Member States assesses the application of Part II with respect to its own territory (The European Parliament and the Council of the European Union, 2014). The reporting Member State and each Member State concerned then notify the manufacturers of their conclusion via CTIS. ATMPs, including PSC-derived products, are reviewed for regulatory approval by the European Medicines Agency (EMA); however, the EMA has no authority over clinical trials. The EMA is responsible for maintaining CTIS, and the European Commission (EC) oversees the implementation of the Clinical Trials Regulation (European Medicines Agency, 2022).

As in the US, clinical trials/research must be conducted in accordance with ICH GCP guidelines, regardless of whether they are non-commercial or commercial. Considerations relating to clinical trial designs and the quality of investigational ATMPs (iATMPs) are stated in the GCP for ATMPs developed in the EU (Table 1, #16) (European Commission, 2019). When conducting clinical studies on ATMPs, an Investigational Medicinal Product Dossier (IMPD) should be prepared. The IMPD is a summary of the investigational drug and describes the drug substance (DS), drug product (DP), device, etc., using the European Directorate for the Quality of Medicines & Health Care standard terms. The DS is defined as the processed starting material used in manufacturing and is associated with information on the name, the type of modality, and the mode of action. The DS provides most of the information on ATMPs. The DP provides information about the route of administration, the sterility, and the dilution of the ATMPs. For a particular iATMP, the starting material, active substance, and final product may be closely related or nearly identical. If possible, active, intermediate, and final products should be identified. The IMPD should also describe the process validation performed and summarize the key points relevant to the understanding of the selected product development approach (Centre for Advanced Medical Products, 2019).

##### Exception systems for the use of ATMPs

For ATMPs to be marketed in the EU, their efficacy, safety, and quality need to be scientifically evaluated for approval review, which is performed by the EMA under the delegation of the EC. The Committee for Medicinal Products for Human Use (CHMP) is responsible for the regulatory review of drugs and medical devices within the EMA; however, as the evaluation of ATMPs is more specialized and involves multidisciplinary perspectives compared with that for conventional drugs and medical devices, the Committee for Advanced Therapies (CAT) was organized as an advisory body for the CHMP. An approval review is conducted by the CHMP based on the evaluation of efficacy, safety, and quality from the CAT. The EC then makes an approval decision based on an evaluation letter prepared by the CHMP. To resolve these requirements, evaluate ATMPs within the EU, and prompt their marketing, the efficacy, safety, and quality of ATMPs are evaluated directly by the CAT. Even ATMPs that are to be distributed in only one member country must be evaluated by the CAT and reviewed by the CHMP.

In the EU, the PRIME program supports the development of drugs for unmet medical needs. PRIME is designed to enhance networking and promote early meetings with developers of promising drugs, with the aim of strengthening support for the development of drugs to treat currently unmet medical needs. This program is expected to optimize the development plan, accelerate the evaluation of new drugs, and make them more readily available to patients. The eligibility of ATMPs for PRIME is determined according to the extent to which they can be applied to unmet medical needs, their expected efficacy, and their improvement over existing treatments and methods. In addition to the data on clinical activity, a summary of all available safety data obtained in non-clinical and clinical settings should also be provided (Table 1, #14) (EMA/CHMP, 2015). ATMPs that qualify for PRIME may be eligible for accelerated assessment with a shorter review period at the time of regulatory approval (European Medicines Agency (c)). In addition, ATMPs are exempt from review by the EMA if there are no other approved drugs for particular conditions with high unmet medical needs and the product is produced non-repetitively at a single hospital within the same member country for a specific patient population (The European Parliament and the Council of the European Union, 2007). This type of approval is called “Hospital Exemption”; however, even products eligible for the Hospital Exemption must be reviewed and approved for manufacturing, quality, and pharmacovigilance by the agency in the country of production and distribution and be produced in good manufacturing practice (GMP)-authorized sites. A recommendations and position paper for the use of Hospital Exemptions is issued from ARM (which involves 350+ members worldwide and 70+ members across 15 European countries) and the European Association for Bioindustries, respectively (Alliance for Regenerative Medicine, 2020; European Association for Bioindustries, 2020). The “Specials” Exemption is another system of exceptional use for specific patients (The European Parliament and the Council of the European Union, 2001b; Medicines and Healthcare products Regulatory Agency, 2014). The Hospital Exemption is only applicable to ATMPs, whereas the “Specials” Exemption covers all medical products including ATMPs (Mahalatchimy and Faulkner). The Specials products are produced in compliance with the GMP by manufacturers granted by the Licensing Authority, and their use is not limited to hospitals. The Specials products can be prescribed by doctors or dentists, supplementary prescribers (such as an appropriately qualified nurse or pharmacist), and others, and this differs from market authorization (Medicines and Healthcare products Regulatory Agency, 2014). As a Hospital Exemption is granted for ATMPs with no demonstrated safety and efficacy performance in some EU countries, there may be concerns about the safety and efficacy of the product, which may not have been confirmed (Cuende et al., 2022). A warning against the use of unproven cell therapies was issued by the EMA in 2020 (EMA/CAT, 2020). Therefore, although Hospital Exemptions and Specials humanely provide patients with opportunities to access ATMPs, these systems should not be used to just avoid the high costs of a clinical trial.

##### Guidelines for quality, safety, and efficacy evaluation

In addition to the general guidelines for human cell-based medicinal products, a reflection paper on specific aspects related to marketing authorization applications for stem cell-based medicinal products has been issued by the EU (Table 1, #10 and #12) (EMA/CHMP, 2008a; EMA/CAT, 2011). The points to consider for quality (including manufacturing) as well as non-clinical and clinical studies are described in this reflection paper. In particular, the tumorigenicity of undifferentiated PSCs is mentioned in the sections on “Quality Considerations” and “Non-Clinical Considerations.” In addition, information on the *in vivo* fate of ATMPs in clinical settings is included in the section on “Pharmacokinetics,” which encourages the development and validation of new, non-invasive methods for tracking cells during clinical trials. On the assumption that ATMPs become part of the patient’s body, unlike conventional drugs and medical devices, EU regulations focus on follow-up and risk management to determine the efficacy of ATMPs and their associated adverse effects. Therefore, the applicant is required to provide details about follow-up and post-marketing surveillance in addition to a risk management plan. The “Guideline on safety and efficacy follow-up and risk management of advanced therapy medicinal products” issued in the EMA provides details for the post-marketing follow-up and risk management of ATMPs (Table 1, #11) (EMA/CHMP, 2008b).

For ATMPs derived from human cells and tissues, it is necessary to comply with Directive 2004/23/EC (The European Parliament and the Council of the European Union, 2004) with respect to donors and processes such as cell preparation/processing, storage, and transfer. In addition, the guidelines on GMP for ATMPs were established to regulate the manufacturing and quality control specific to ATMPs in the EU (Table 1, #15) (European Commission, 2017). In accordance with the GMP, the WHO and ISO have also produced international guidance, the Technical Report Series 1044 – 56^th^ report of the WHO Expert Committee on Specifications for Pharmaceutical Preparation (World Health Organization, 2022a), and ISO/IEC 17025:2017 – General requirements for the competence of testing and calibration laboratories (International Organization for Standardization, 2017), respectively.

#### Japan

##### Product classification in Japan

Under The Act on Securing Quality, Efficacy and Safety of Products Including Pharmaceuticals and Medical Devices (Pharmaceuticals and Medical Devices Act, PMD Act), which regulates products intended for marketing, cell-processed products and products for gene therapy are collectively referred to as “regenerative medical products,” which is a product type that is independent from that of drugs and medical devices. Products composed of living human or animal cells are classified as regenerative medical products in accordance with the presence or absence of cell processing and non-homologous use. Unlike in the US and the EU (and UK), the genetic modification of cells is considered to be a type of cell processing; therefore, genetically engineered cells for *ex vivo* gene therapy, for example, CAR T cell products, are classified as cell-processed products in Japan and not as products for gene therapy. PSC-derived products are also classified into cell-processed products. Under The Act for the Safety of Regenerative Medicine (RM Safety Act, ASRM), which is applied to regenerative/cellular therapies conducted as medical practice solely under the discretion of medical practitioners or as non-commercial clinical research, human or animal cells that are cultured or otherwise processed and are not classified as regenerative medical products under the PMD Act are called “specified processed cells.”

##### Commercial clinical trials and non-commercial clinical studies

As described above, in Japan, the law applicable to CTPs with manufacturing/marketing authorization or commercial clinical trials for the purpose of obtaining manufacturing/marketing authorization differs from that applicable to medical practices using specified processed cells (namely, CTPs without manufacturing/marketing authorization) solely at the discretion of the medical practitioners or to non-commercial clinical studies on specified processed cells. Commercial clinical trials are regulated by the PMD Act and must be conducted in compliance with the GCP. If an application for marketing authorization is submitted, it is reviewed by the Pharmaceuticals and Medical Devices Agency (PMDA) and the Ministry of Health, Labor and Welfare (MHLW). In contrast, medical practices using specified processed cells based solely on physicians’ discretion and non-commercial clinical studies on specified processed cells are out of the scope of the PMD Act, and they do not need to meet the data quality assurance-related requirements of the GCP. However, they must comply with the same ethics-related requirements as the GCP under the regulations of the RM Safety Act. Medical practices using specified processed cells have three classes in terms of the risk not conducting an appropriate safety assessment. Medical practices using PSC-derived specified processed cells are classified into “Class 1 Regenerative Medicine,” which are deemed the most difficult to assess for safety. Although the MHLW may provide opinions about the plans for non-commercial clinical studies on PSC-derived specified processed cells under the RM Safety Act, the reviews are not conducted by the PMDA but by institutional committees called Specified Accredited Committees for Regenerative Medicine. The RM Safety Act also provides the standards for institutions and cell processing facilities to ensure the safety of regenerative medicine and cell therapy.

##### Manufacturing/marketing authorization in Japan

In the process of manufacturing/marketing authorization for regenerative medical products, there is a conditional and term-limited approval system that allows for early patients’ access, in addition to the regular approval system. Based on the application data, PMDA and MHLW (not a pharmaceutical company) determine whether a term-limited approval system is applied to the regenerative medical product. In a case in which data about the efficacy, safety, and quality of CTPs are fully demonstrated, the CTPs can be approved via the regular approval system. In the conditional and term-limited approval system, the MHLW may grant approval with necessary conditions that ensure its proper use and a term limit not exceeding 7 years, if the following criteria are met: (1) the regenerative medical product pertaining to the application is not homogeneous; (2) the product is presumed to have the efficacy pertaining to the application; (3) the product is presumed to have value for use as a regenerative medical product because it does not have a significantly harmful effect in comparison with the efficacy, effectiveness, or performance pertaining to the application. For regenerative medical products granted conditional and term-limited approval, it is necessary to plan and conduct a post-marketing study to verify the efficacy and safety and to re-submit the application to obtain official approval within 7 years; this process differs from that of the regular approval system in Japan. Although any regenerative medical products granted conditional and term-limited approval are covered by public medical insurance, the data need to be evaluated for all patients using the product in question. It should be noted that it is impossible to conduct a randomized controlled study after marketing. Therefore, MHLW encourages that post-marketing data be consolidated in a nation-wide database called National Regenerative Medicine Database, which was established by PMDA and has been operated by the Japanese Society for Regenerative Medicine (Okada et al., 2018), to be evaluated with the cooperation of relevant academic societies (MHLW/PFSB/ELD, 2017).

##### Guidelines for quality, safety, and efficacy evaluation

In Japan, various notifications have been issued on CTPs, and their general descriptions are similar to the guidelines used in the US and the EU. To make it easier for developers, the MHLW has issued five guideline documents that describe the basic technical requirements for ensuring the quality and safety of CTP for each type of starting cell. Three of the five documents specialize in PSC-derived CTPs (Table 1, #17–19) (MHLW/PFSB, 2012a, 2012b, 2012c; Hayakawa et al., 2015a, 2015b, 2015c). These three guidelines have a common basic content, although the allogeneic guidelines emphasize HLA typing and viral and bacterial infections associated with allogeneic PSCs. Understanding the genetic background of the donor is important when establishing allogeneic iPSC lines and may become much clearer when there is an understood hPSC-based product type. The difference between the guidelines for allogeneic iPSCs and ESCs is that, as ESCs are generated from the inner cell mass of a blastocyst, the guideline for ESCs describes ethical considerations, eligibility of the person or medical institution that collects the cells, and the validity of the method used to prepare fertilized embryos. It is also necessary to understand the genetic background when establishing ESC lines, but this applies to male and female donors for producing *in vitro* fertilized embryos. Recently, for products whose clinical application or marketing are anticipated, evaluation guidelines have been issued as monographs based on the characteristics of the products, which are designed to encourage product developers and regulatory reviewers to share points to consider for discussions. (Table 1, #20, #21, #23, #25, and #27) (MHLW/PFSB/ELD/OMDE, 2013; MHLW/PFSB/ELD/OMDE, 2014; MHLW/PSEHB/MDED, 2016a; MHLW/PSEHB/MDED, 2021; MHLW/PSEHB/MDED, 2023).

As a ministerial ordinance on the standards for manufacturing and quality controls of regenerative medical products, the Good gene, Cellular, and Tissue-based products manufacturing Practice (GCTP) ordinance was issued (MHLW, 2014). The GCTP ordinance primarily considers structural facilities, manufacturing controls, and quality control. Quality risk management, verification, and product quality review have been stipulated in the GCTP but not in the GMP. In general, validation is defined as an action of proving and documenting that any process, procedure, or method actually and consistently leads to the expected results (World Health Organization, 2022a). Validation is required for the manufacturing of pharmaceutical products; however, it is difficult to identify factors causing variations in cell preparation. In this case, the achievement of expected quality should be verified for each production batch by reviewing the written procedures, protocols, records, and reports, which are documented. This is called verification. In Japan, non-commercial clinical studies and medical practices conducted under the RM Safety Act also need to be compliant with the similar GCTP for the manufacturing and quality controls of specified processed cells.

A variety of biological materials can be used to manufacture regenerative medical products. In Japan, the Standards for Biological Raw Materials was established in 2003 (MHLW, 2018), which apply to biological raw materials derived from animals and human used for manufacturing of CTPs as well as to pharmaceutical products. With respect to the terminology used for materials employed in manufacturing in the EU, raw materials are considered to be the materials used during the manufacture of the active substance (e.g., culture media, growth factors), and they are not intended to form part of the active substance for ATMP (The European Parliament and the Council of the European Union, 2001b). The materials forming an integral part of the active substances are considered to be “starting materials.” The positioning of a starting material varies depending on the nature of the product. In the United State Pharmacopeia, raw materials are defined as all materials used in the manufacture of cell and gene therapy products (such as cells, tissues, matrices, media, and buffers), whereas ancillary materials are a subset of raw materials that come in contact with the cell or tissue product, but they are not intended to be part of the final product. Therefore, the terminology used to describe materials for CTP manufacturing is confusing because of the inconsistent classification, naming, and labeling of intended use between regions (Solomon et al., 2016). Biological manufacturing-related materials are considered potential hazards (risk factors) in the following two cases: (1) when biological manufacturing-related materials are present or remain in the final product in a significant amount that may cause undesirable health problems in patients and (2) when there is a possibility (including a theoretical possibility) that biological manufacturing-related materials may be contaminated with viruses or prions that may cause undesirable health problems in patients administered the final products. On the other hand, products that are already approved as pharmaceuticals or additives listed in Japanese official standards, such as the Japanese Pharmacopoeia and the Standards for Biological Raw Materials, and fractionated plasma products derived from domestic blood donors and their equivalents are generally not considered hazards and are not subject to evaluation unless the approved dosage is exceeded. The Standards for Biological Raw Materials require a great deal of information, including donor screening, viral safety test results, and donor traceability, which are linked to issues with infectious agents. However, in many cases, manufacturers of raw materials are often unable to provide full information about their quality because some raw materials are used only for research purposes. In addition, the appropriate information may not have been gathered for existing batches, or it is too difficult and costly to establish, and the raw material manufacturer has no commercial inducement. For example, their main income may be derived from supplying their product for other purposes, such as research or food production; in this respect, hydrolysates used in culture media are the by-product of dairy-based foods. To improve such issues associated with biological raw materials, it may be beneficial to implement the following: 1) collaborate with the raw material producer to establish a type of "master drug file" for the material, 2) review the necessity for additional product or raw material testing regimes based on a description of the manufacturing process and raw materials, and their origins, and 3) conduct a site inspection of the raw material producer.

A case-by-case approach is required to develop CTPs, resulting in no uniform standards and many uncertainties. With this background, the PMDA has introduced the current approach to quality control, non-clinical safety evaluation, and points to consider in clinical studies (Table 1, #22) (MHLW/PSEHB/MDED, 2016b) based on its experience in face-to-face consultation and review concerning CTPs with characteristics that are significantly different from those of pharmaceuticals and medical devices.

Transformed cells and residual undifferentiated PSCs as product impurities are potential tumorigenic hazards specific for CTPs. A guideline has been issued that proposes representative examples of testing methods for detecting undifferentiated PSCs and transformed cells intermingled with human CTPs as well as points to consider when selecting testing methods for evaluating the quality and safety of particular human CTPs (Table 1, #24) (MHLW/PSEHB/MDED, 2019). In this guidance document, it is noteworthy that the section of tumorigenicity-related tests for human ESC/iPSC-based products is separated into three parts, which depend on the purpose of testing for CTPs: (1) quality control of cell substrates, (2) quality control of intermediate/final products during manufacturing processes, and (3) non-clinical safety assessment of final products. On the other hand, another guidance document provides several points to consider for the tumorigenicity assessment of unlicensed PSC-derived processed cells in non-commercial clinical studies, which are regulated by the RM Safety Act (Table 1, #26) (MHLW/HPB/RDD, 2021). This document states that to provide patients with therapies using PSC-derived processed cells as safely and rapidly as possible, it is necessary to collect and accumulate scientific data in non-commercial clinical studies for future development of regenerative medicine and cell therapy, particularly those that accumulate data on the genomic instability of products and the technical issues associated with testing methods.

### Global technical standardization and regulatory harmonization/convergence

**Figure 1:**
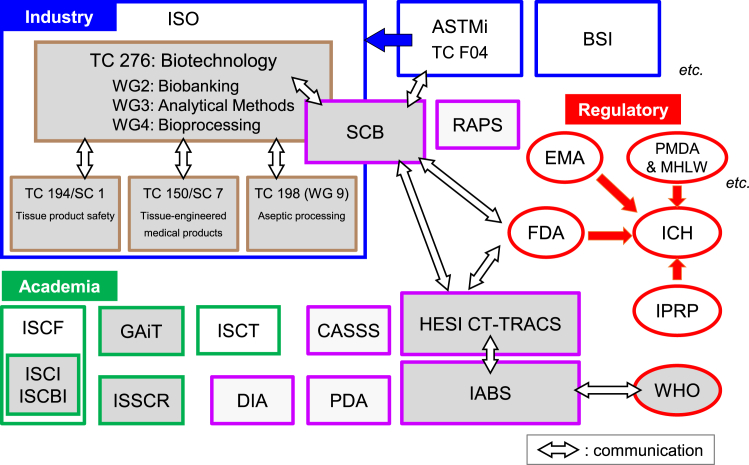
Platforms for discussions on international regulatory harmonization/convergence and technical standardization of cell-based therapeutic products Representative platforms/consortia related to international regulatory harmonization/convergence and technical standardization of pluripotent stem cell-based therapeutic products are shown (open, no discussion on international harmonization and standardization; filled, discussion on international harmonization and standardization). *Industry*: ISO, International Organization for Standardization; ASTMi, American Society for Testing and Materials International; BSI, British Standards Institution; SCB, Standards Coordinating Body for Cellular/Gene and Regenerative Therapies. *Academia*: ISCT, International Society for Cell & Gene Therapy; ISSCR, International Society for Stem Cell Research; GAiT, Global Alliance for iPSC Therapies; ISCI, International Stem Cell Initiative; ISCBI, International Stem Cell Banking Initiative; ISCF, International Stem Cell Forum/Foundation; DIA, Drug Information Association; CASSS, California Separation Science Society. *Regulatory*: FDA, Food and Drug Administration; EMA, European Medicines Agency; PMDA, Pharmaceuticals and Medical Devices Agency; MHLW, Ministry of Health, Labor and Welfare; ICH, International Council for Harmonization of Technical Requirements for Pharmaceuticals for Human Use; IPRP, International Pharmaceutical Regulators Program; WHO, World Health Organization. *Others*: HESI CT-TRACS, Committee for Cell Therapy-Tracking, Circulation & Safety, Health and Environmental Sciences Institute; IABS, International Alliance for Biological Standardization; DIA, Drug Information Association; CASSS, California Separation Science Society; RAPS, Regulatory Affairs Professional Society; PDA, Parenteral Drug Association.

Regulations for CTPs have not yet been harmonized. Discussions on standardization and regulatory harmonization/convergence are ongoing in various consortia involving members from industry, academia, and regulations (Figure 1). The following is an overview of the scope and efforts of representative consortia.

#### Standardization of PSCs as raw materials for CTPs

##### The International Stem Cell Initiative

In the 2000s, human PSCs were new, and their properties were not yet fully understood. In addition, the results reported by laboratories often differed. Recognizing these challenges, researchers organized the first meeting of the International Stem Cell Forum (ISCF; later renamed the International Stem Cell Foundation) in Paris in 2003 (Times Higher Education, 2003). Twenty-two countries participated in the Forum, including the US, the UK, France, Germany, and Japan, and they proposed the launch of a project for collecting and comparing PSCs from around the world, with the aim of determining the common characteristics of human PSCs. Funded by the ISCF, the International Stem Cell Initiative (ISCI) was initiated in the autumn of 2003 with the goal of sharing knowledge about the characteristics of human PSCs and stimulating the development of their applications (Andrews et al., 2005). To date, the ISCI has compared the expression of undifferentiated and differentiated markers in the undifferentiated state and in the embryoid state of a number of human PSC lines (ISCI-1) (International Stem Cell Initiative, 2007), examined their profile of genomic variation by long-term *in vitro* culture (ISCI-2) (International Stem Cell Initiative Consortium, 2010; Närvä et al., 2010), and evaluated the performances of methods to measure pluripotency and differentiation potential of human PSCs (ISCI-3) (International Stem Cell Initiative, 2018). In addition, the ISCI Steering Committee has recently published a collective perspective on the current understanding of the genetic and epigenetic variations that occur in human PSCs (Andrews et al., 2022).

##### The International Stem Cell Banking Initiative

Based on the results of the ISCI-1 activities, the International Stem Cell Banking Initiative (ISCBI) was launched in 2007, which consists of researchers from stem cell banks from 28 countries around the world, including the US, France, Germany, Japan, and others, with the UK Stem Cell Bank as its core. The ISCBI supports the international technical standardization of PSCs through discussions on cell line procurement, cell banking systems, optimization of storage methods, advanced aseptic processing, and quality control systems, including virus testing (International Stem Cell Banking Initiative). Reports and results from ISCBI activities have been published in papers and other media (International Stem Cell Banking Initiative, 2009; Crook et al., 2010; Andrews et al., 2015; Stacey and Healy 2021; Kim et al., 2022).

##### Pluripotent stem cell standards initiative task force on the International Society for Stem Cell Research

The International Society for Stem Cell Research (ISSCR) is a non-profit stem cell research society that holds a forum for sharing information on PSCs in collaboration with stem cell societies in various countries. The Pluripotent Stem Cell Standards Initiative Task Force of the ISSCR summarizes information on the quality of PSCs that is recommended when submitting papers to scientific journals, based on the evaluation of cell line characteristics. The project has drafted a guidance document for basic and genomic characterization of PSCs, identifying undifferentiated stem cells, assaying pluripotency, and stem cell-based model systems, which will be finalized and published in the first half of 2023 (International Society for Stem Cell Research).

##### The Global Alliance for iPSC therapies

The Global Alliance for iPSC Therapies (GAiT) is an international consortium of interested partners, some of which are resource centers that facilitate the therapeutic use of immunogenetically matched and clinical-grade iPSCs for the benefit of patients worldwide. The scope of GAiT is to achieve a consensus on donor selection, screening criteria, manufacturing, and quality parameters and to gain agreement on the quality standards with regulators. In addition, GAiT is involved in the development of an external quality assurance scheme for iPSC lines intended for clinical use (Sullivan et al., 2020).

##### Human pluripotent stem cell registry

The Human Pluripotent Stem Cell Registry (hPSCreg) is an international resource that registers and collects the standard properties of hPSC lines (Mah et al., 2020). The hPSCreg platform was established to provide a database for clinical studies involving PSC-derived products (Kobold et al., 2020); the database acts a regulatory tool of the EC and ensures that all iPSC lines used in EC-funded research are ethically sourced and available for public research.

##### ISO TC276 working group 2

The ISO establishes international definitions, standards, and guidelines. TCs has been established for discussing a variety of items in the ISO. Regenerative medicine is discussed in TC276: Biotechnology, which is divided into five working groups (WG1: Terminology, WG2: Biobanking, WG3: Analytical Methods, WG4: Bioprocessing, and WG5: Data Processing and Integration). Other relevant TCs include ISO/TC 150/SC 7 – tissue-engineered medical products, which discusses the standardization of methods for evaluating the efficacy of regenerative medicine products in combination with scaffolds; ISO/TC 194/SC 1 – tissue product safety, which discusses biological evaluation methods for the safety of medical materials and devices; and ISO/TC 198/WG9 – aseptic processing, which discusses the processes of aseptic manipulation for regenerative medicine products.

ISO/TC 276/WG2 discusses standardizations related to biobanks and bioresources, including human cells, animal cells, plants and seeds, and bacteria. Standards of PSC stocks used in research are pursued in WG2, and these were recently published as ISO 24603:2022 (International Organization for Standardization, 2022a). One of the future goals of WG2 is to standardize PSC stocks for clinical use.

#### Standardization of technologies for CTPs

##### ISO: TC276 WG1/WG3/WG4

Working groups 1, 3, and 4 in ISO TC276 are related to technologies for CTPs. In WG1, terminology is defined for use as a common language among countries in relation to trade. In WG3, analytical methods for measurements related to cells and nucleic acids are standardized. Discussions have been held on available indicators for accurately evaluating equipment and cell counting methods that are difficult for SI-traceable measurement, as well as the requirements and measurement techniques used to identify cells. The documents already published include the following: “ISO 23033:2021 Biotechnology – Analytical methods – general requirements and considerations for the testing and characterization of cellular therapeutic products,” which describes the general approach used to determine methods for evaluating the quality of cell therapy products and considerations for determining the checklist quality of CTPs (International Organization for Standardization, 2021a), and “ISO 20391-1:2018 Biotechnology – cell counting – Part 1: general guidance on cell counting methods,” which describes cell counting methods (International Organization for Standardization, 2018). WG4 standardizes bioprocessing, and this is particularly relevant for regenerative medicine. This WG has worked on the standardization of raw materials, transportation, equipment, and stabilization of cell manufacturing, and the following documents have been published: ISO 21973:2020 Biotechnology – general requirements for the transportation of cells for therapeutic use; ISO/TS 23565:2021 Biotechnology – bioprocessing – general requirements and considerations for equipment systems used in the manufacturing of cells for therapeutic use; ISO 20399:2022 Biotechnology – Ancillary materials present during the production of cellular therapeutic products and gene therapy products (International Organization for Standardization, 2020, 2021b, 2022b).

In addition, documents associated with 18 other topics are currently being developed in ISO 276 (International Organization for Standardization). For example, there are “ISO/WD 18162 Biotechnology – Biobanking – Requirements for human neural stem cells derived from pluripotent stem cells,” which is the standard for biobanking of iPSC-derived hNSCs used for research and development in the life science field, but not for *in vivo* human use, clinical use, or therapeutic purposes, and “ISO/CD 8472-1 Biotechnology – Data interoperability for stem cell data – Part 1: Framework” and “ISO/AWI 8472-2 Biotechnology – Data interoperability for stem cell data – Part 2: Key characteristics of stem cell data” which are standards that provide a framework for data interoperability of stem cells that are being developed.

##### The Standards Coordinating Body for gene, cell and regenerative medicines and cell-based drug discovery

The Standards Coordinating Body for gene, cell and regenerative medicines and cell-based drug discovery (SCB) is a non-profit organization, which was established in the US at an initiative of the Alliance for Regenerative Medicine (ARM) and other regenerative medicine stakeholders and industry to promote standardization in the nascent regenerative medicine industry. The long-term goal of the SCB is to support efficient and effective product development and review by assisting in the development of standards available for regulatory review and improving the cost, time, and resources for product development and approval (Standards Coordinating Body). The SCB provides coordination to promote the development of standards for regenerative medicine manufacture, and it continues to participate in ISO TC276 discussions, as well as FDA (CBER) and NIST.

#### Global regulatory harmonization/convergence

##### International Council for Harmonization of Technical Requirements for Pharmaceuticals for Human Use

The objectives of ICH are as follows: (1) to ensure that patients have timely and continuous access to new pharmaceutical products; (2) to avoid unnecessary duplication of clinical studies in humans; (3) to ensure efficient development, registration, and manufacturing of safe, effective, and high-quality pharmaceutical products; and (4) to promote public health by facilitating international harmonization of technical requirements that help reduce animal testing without compromising safety and efficacy. The ICH guidelines cover all aspects of pharmaceutical products, including ICH Q (1–14) for quality, ICH S (1–12) for non-clinical (safety), ICH E (1–20) for clinical (efficacy), and ICH M (1–14) for multidisciplinary evaluation. Of these guidelines, Q5A, Q5D, Q5E, Q6B, and S6, while CTPs are not officially within their scopes, are those that provide very useful information about CTP development and manufacturing. However, it is difficult to uniformly apply them to CTPs, as in the case with conventional medicines. At the 12th Summit of Heads of Medicines Regulatory Agencies in 2017, recognizing the need to implement regulations that appropriately reflect the characteristics of regenerative medicine products and their international harmonization, an agreement was made to promote international regulatory harmonization for regenerative medicine products by utilizing existing international frameworks (WHO, ICH, etc.). However, no global regulatory guidelines have been established for the use of CTPs to date.

##### World Health Organization

The World Health Organization (WHO) is an organization that was established to help countries cooperate with each other to promote and protect the health of all people, and it has a role in the standardization of biologicals via its Expert Committee on Biological Standardization (ECBS). The WHO ECBS has currently progressed beyond well-established biologicals to cell-based medicines. With the aim of explaining WHO’s current thinking on the regulation of cellular and gene therapy products, promoting convergence, and encouraging member states to strengthen their regulatory systems for the regulation of cellular and gene therapy products, WHO recently adopted the “WHO approach toward the development of a global regulatory framework for cell and gene therapy products” (World Health Organization, 2022b). This document is not intended to be a comprehensive overview of the regulatory requirements for cellular and gene therapy products or the different regulatory frameworks that currently exist in different jurisdictions. The purpose of this document is to outline some of the basic principles that are important for appropriate regulatory oversight for different types of cellular and gene therapy products. In the future, WHO will develop more comprehensive written guidance on specific topics relevant to the regulation of cellular and gene therapy products, as needed.

#### Other platforms

##### The International Alliance for Biological Standardization

The International Alliance for Biological Standardization (IABS) was founded in 1955 to improve the quality and regulation of biological products from human and animal origin in association with the WHO, manufacturers, and regulatory bodies. The IABS consists of members in over 50 countries (International Alliance for Biological Standardization), and meetings have taken place in 2014, 2016, 2018, and 2020. Notably, the meeting in 2018 meeting was focused on the manufacture of human pluripotent stem cell-based products (Abbot et al., 2018). The IABS collaborated with the WHO to publish a white paper on scientific considerations when evaluating cell therapy products (Petricciani et al., 2017).

##### The Cell Therapy-Tracking, Circulation, & Safety Committee, and the Health and Environmental Sciences Institute

The Health and Environmental Sciences Institute (HESI) functions as a global platform for industry, academia, and government to engage in scientific discussions on methods that evaluate quality and safety. Methods to evaluate the tumorigenicity of CTPs and analyze their biodistribution have been studied by the Cell Therapy-TRAcking, Circulation, & Safety (CT-TRACS) Committee. In Japan, the Japan Agency for Medical Research and Development has been supporting a public-private partnership initiative for the multisite evaluation study on analytical methods for non-clinical safety assessment of human-derived regenerative medical products (MEASURE), which aims to validate methods for assessing the tumorigenicity of CTPs. The data from the MEASURE project are shared with HESI CT-TRACS members to discuss the validity and points to consider for the test methods. Their cooperation is expected to be helpful for the international standardization and regulatory harmonization/convergence of test methods for CTPs in the future (Sato et al., 2019; Kamiyama et al., 2021; Watanabe et al., 2021).

## Conclusion

In this paper, we introduce regulations related to PSC-derived CTPs, mainly those of the US, EU, and Japan, and the efforts made to standardize various regulatory platforms (Figure 1 and Table 2). In summary, PSC-derived CTPs are currently evaluated using the general approach applied to CTPs. In the US, although the FDA’s Advisory Committee has provided points to consider for preclinical safety testing and patient monitoring for ESC-derived CTPs, no official guidelines have been issued, and thus far, CTPs derived from stem cells are reviewed within the framework of 351 HCT/Ps. Conversely, in the EU and especially in Japan, guidelines for CTPs derived from stem cells have been specifically issued, addressing tumorigenicity, one of the concerns with PSC-derived CTPs. Referring to hESC guidelines available for the development of CTPs in the UK, the UK Stem Cell Bank was established in 2003 to facilitate the sharing and use of quality-controlled human stem cell lines by clinical and research communities. UK documentation on the use of hESCs was published by the “Steering Committee for the UK Stem Cell Bank and the Use of Human Embryonic Stem Cells,” which provides guidance and assistance on best practice to those working with stem cell lines (UK Stem Cell Bank steering committee, 2010).

**Table 2:** Regulation of cell-based therapeutic products in the United States, the European Union (and the United Kingdom), and Japan

	US	EU (and UK)	Japan
Classification of PSC-derived CTPs	351 HCT/P	Advanced Therapy Medicinal Product (ATMP)	Cell-Processed Product
Product type	Biologics or Medical Devices	Medicinal Products	Regenerative Medical Products
Regulatory authority	FDA	EMA (MHRA in UK)	MHLW and PMDA
Compliance with GCP in clinical trials	essential	essential	essential in commercial clinical trials
Good Practice(s) for Quality and Manufacturing Controls	cGMP (for biologics) or QSR (for medical devices)	GMP for ATMPs	GCTP
Conditional Marketing Authorization with Putative Efficacy	RMAT/HDE	Hospital Exemption (Article 28 of Regulation 1394/2007/EC)	Conditional and Term-limited Approval
Use of unlicensed products	Federal regulations prohibit manufacturers from introducing unapproved 351 HCT/Ps into interstate commerce.	Specials (Article 5 (1) of Directive 2001/83/EC)	Specified Processed Cells under the RM Safety Act

As described above, tumor formation of residual tumorigenic cells is a concern for the clinical application of PSC-derived products. However, the threshold levels for residual tumorigenic cells, including undifferentiated hiPSCs, in tumor formation attributed to CTPs are ill-defined. As the properties of CTPs and the performance of testing methods determine the threshold, the possibility of tumor formation from a single tumorigenic cell cannot be completely excluded. In practice, a 50% tumor producing dose (TPD_50_) from positive control iPSCs was reported as 132 and 631 cells under the condition that is suitable for iPSC survival using *in vivo* testing with severely immunodeficient NOG mice (Kanemura et al., 2014; Yasuda et al., 2018). Therefore, specifications for residual tumorigenic cells in CTPs should be defined as being “less than the detection limit using the testing method” unless justified otherwise. *In vivo* testing would also be required to evaluate the tumorigenicity of transplanted CTPs in the microenvironment, especially for PSC-derived products and other types of CTPs with limited clinical experience. In such a case, the selection of animal models and species requires scientific justification. The use of genetically immunodeficient animals and humanized animals is recommended in the US and EU to monitor tumor formation over the long-term (Table 1, #5 and #12) (FDA/CBER, 2013; EMA/CAT, 2011). In Japan, the use of severely immunodeficient NOG mice and NSG mice is preferred to that of nude mice, with respect to the easy engraftment of xenogeneic cells (Table 1. #24) (MHLW/PSEHB/MDED, 2019). Also, several *in vitro* tests have shown to be comparable to *in vivo* tests using such severely immunodeficient mice at least in terms of the detection limit for tumorigenic cellular impurities in intermediate or final products. The FDA Modernization Act 2.0 was finally approved in the US, and this aims to improve the process involved in approving drugs and commits to significantly reducing the use of animals in laboratory testing (FDA Modernization Act 2.0, 2022). Therefore, clinical trial leaders will use animal trial alternatives instead of traditional animal modeling for drug development in non-clinical studies. In the EU, the document “Guideline on the principles of regulatory acceptance of 3Rs (replacement, reduction, refinement) testing approaches” has already been issued by the EMA (EMA/CHMP/CVMP, 2016). In this context, as critical quality attributes of PSC-derived CTPs associated with *in vivo* tumor formation are also gradually revealed, *in vitro* testing would be more useful and reasonable for tumorigenicity evaluation, as this would reduce the number of animals used in *in vivo* tumorigenicity testing in the future.

In the regulations of the three (or four including UK) ICH founding states, the risk-based approach as a regulatory principle is more emphasized for CTP than for other drugs. Each country or region has developed its own expedited approval process for CTPs that differs from that for conventional pharmaceuticals. Some of these systems allow patients early access to CTPs when safety is assured and putative efficacy is confirmed. Although they require continuous evaluation, such as post-marketing surveillance, these programs can address the needs of patients with diseases for which no other treatments are available and promote product development. Various consortia have recently discussed the move toward standardization of CTPs regulation in accordance with their respective scopes. Indeed, the US is focusing on standardization as a national policy, so the importance of standardization is expected to increase in the future. Standardization would be beneficial for both CTP development and regulation, which would promote their clinical application. Patients would have early access to CTPs if standardization resolved issues related to drug lag. The ICH is responsible for the harmonization of pharmaceutical regulations, but it has not yet actively worked toward obtaining regulatory harmonization/convergence of CTPs. Hopefully, a consensus on regulations related to CTPs, including PSC-derived products, will be formed through the ICH in the future. As the final products of PSC-derived CTPs are diverse, we suggest that the commonalities and differences between the quality properties of products should be defined, and this will require a flexible approach to issues demonstrated by scientific findings. It is necessary to establish a system for sharing data and their accompanying interpretations and perceptions on a global scale as data related to PSC-derived CTPs are accumulated.

## Author contributions

Y.S. and A.U. provided conception and obtained funding for this paper; T.H. surveyed the updates of regulations and consortia related to CTPs and wrote the first draft in collaboration with S.Y. All authors reviewed the manuscript and approved its final version.
